# “Embedding” and “pulling back”: Spatial transformations and urban assimilation of migrant elderlies following their children

**DOI:** 10.3389/fpubh.2022.1009274

**Published:** 2022-11-21

**Authors:** Yaxiong Bao, Juanmei Tao, Qian Liu

**Affiliations:** ^1^School of Journalism and Culture Communication, Zhongnan University of Economics and Law, Wuhan, China; ^2^School of Journalism and Communication, National Media Experimental Teaching Demonstration Center, Jinan University, Guangzhou, China

**Keywords:** migrant elderly, urban assimilation, conceptual framework, health of the elderly, Spatial transformation

## Abstract

**Background:**

Due to the rapid acceleration of social mobility and the shrinking size of families, China has begun to enter a new form of aging society, with an increasing number of migrant elderlies following their children. How to adapt and assimilate into the new living space profoundly affects those migrant elderlies' mental health. Drawing on the spatial framework proposed by Henry Lefebvre, this paper explores the factors affecting urban assimilation of migrant elderlies following their children in China, and puts forward corresponding strategies to promote the urban assimilation.

**Method:**

Using semi-structured interviews and participatory observation, this study conducted a qualitative study among migrant elderlies following their children who lived in a University Community in Wuhan city, Hubei Province from May 2022 to July 2022. During the survey period, we participated in the gathering activities of the migrant elderly five times a week, and conducted semi-structured interviews with 15 migrant elderlies following their children.

**Results:**

Firstly, this study reveals that family assimilation is the foundation of urban assimilation of migrant elderly following their children; Secondly, we could conclude that the urban life of the migrant elderlies are mostly community-based, so it is especially important for them to reconstruct close neighborhood relations and regain the humanity affection of the traditional acquaintance society. Lastly, the fundamental institutional barrier is a significant factor that influences the ability of these migrant elderlies to live a stable urban life. The Chinese government needs to promote a nationwide unified pension and health insurance system, so that the migrant elderlies can enjoy the same benefits as the local elderly residents in the “inflow” area.

## Introduction

According to the data of the seventh national population census, in 2020, the population aged above 60 years old will account for 18.70%, up 5.44 percentage points from a decade ago in China (1). We are witnessing a new phase of aging society, and many aging problems begin to emerge. For example, the difficulties encountered by left-behind elderly or the elderly citizens living alone in their lives have given rise to a series of social problems, such as suicide (2), fraud (3) and low social participation (4), which have imposed new requirements on China's welfare and social governance. Different from western countries, under the influence of the concepts of filial piety culture, raising children for old age, and generation-skipping care, it has become a cultural habitus for the elderlies to live with their children in China. Therefore, more and more elderly people follow their children to migrate to other places. According to *the China Migrant Population Development Report 2018* released by the National Health Commission of the People's Republic of China, the number of migrant elderlies in China has reached 17.784 million, and its proportion in the migrant population is ever growing (5). The “rural-urban” and “urban-urban” population movement have become the major migration patterns of migrant elderlies. Among them, migrant elderlies who move from rural areas with their children, suffer both physical and mental instability in their old age, and face “dual dis-embedding” of social relationships are more vulnerable to structural difficulties. This represents an epitome of today's “aging China”.

As a relatively marginal group in the labor force system, the elderly population used to be of low mobility. Therefore, both Chinese and foreign academics have paid much less attention to migrant seniors than to other migrant populations. Researches on migrant seniors have only increased in recent years as aging societies are spreading globally. In general, studies by Western scholars involve the motivation, decision-making process, and behavioral characteristics of migrating seniors. The push-pull theory of migration (6), life course theory (7), and place-identity theory (8) constitute the main theoretical framework for them to explain the mobility of the elderly population. These studies suggest that the major motivations for the elderly to migrate are sound living environment (9), economic incentives (10), social relationships (11) and elderly services (12). However, under the influence of the urban-rural dual structure, traditional Eastern-style kinship, and the ideas of filial duty, the main drivers of migration for China's elderly population differ significantly from those of Western countries. Family reunion and raising their grandchildren are the most important reasons for them to move to the cities where their children work (13). That's why they are dubbed as “migrant elderlies following their children” and “elderly drifters” by Chinese mass media and sociological researchers, which can reflect their highly dependence on their families and separation from friends or contacts.

Due to changes in the location of elderly healthcare and the social security environment, Chinese migrant elderlies following their children face the problem of “spiritual empty nest” while lack a sense of belonging in the city. For example, studies have shown that low level of acculturation and intergroup contact in the inflow area significantly affect their mental health (14). The difference in the remote reimbursement rate of medical insurance and the electronic clinical consultation system makes it more difficult for the migrant elderlies following their children to seek medical treatment on their own. The expression “invisible men of the city” can best portray their urban life. In essence, all those similar problems can be attributed to the issue of “urban assimilation” of migrant elderlies following their children.

Currently, the Sustainable Development Goals featuring “universal health coverage” and “leaving no one behind” have become a global health issue. In this context, the urban assimilation of migrant elderlies following their children is also a significant dimension of “health”. In the case of China, the problems of survival and spiritual and cultural life of the migrant elderlies following their children, an inevitable outcome of rapid urbanization, are distinctively different from those of other counties. The Chinese government must face these challenges if it aims to improve the assimilation of urbanization. Focusing on the migrant elderlies following their children during the urban assimilation, this study examines the impacts of the institutional space represented by China's basic medical insurance system and the network space based on social interactions from the aspect of spatial theory, and puts forward corresponding strategies to improve the urban assimilation of migrant elderlies to enhance their medical and health care.

## Literature review

### Spatial transformations and mobile society

“Space” is one of the central concepts of social theory and has been discussed for generations. However, early thinkers, represented by Descartes, regarded “space” as one of the absolute “things” (15). It was not until late twenteeth century, with the publication of *the Production of Space* by the French neo-Marxist philosopher Henri Lefebvre, that the social sciences witnessed a remarkable “turn of space”. “Space” becomes a social product and a field with practical significance (16), and plays an important role in modern society. Henri Lefebvre, one of the first scholars to systematically address the sociological theory of space, argues that space is at least three-dimensional, comprising a hollowed-out physical space, a social space stuffed with relationships, and a symbolic mental space. To be specific, physical space mainly refers to the place where people live, the place where functional activities are realized, and the real field that exists objectively. It is visible and tangible, and has geometric scale, clear geographical location and clear boundaries. A few neighborhoods and a community can be classified into physical space. But the boundaries of social space are more blurred. It is the space perceived and utilized by social groups, and also the space dominated by social activities and social organizations. Social space originates from collective life (17), where unique social institutions and regulations, interests, power and contradictions exist. People have to interact within the scope prescribed and allowed by the social institutions, and also have to interact socially within the framework of power fields in order to construct different categories of social relationships and satisfy their own social, spiritual and emotional needs. Spiritual space is the most abstract and subjective category of space with the most blurred boundaries. As the highest level of the space layer, it is the place of personal spiritual and emotional belonging and concerns individual identification. On the whole, these three levels of space do not exist independently, but are embedded and interpenetrated with each other. Together, they constitute the specific fields of human life.

In the twenty first century, with the acceleration of globalization and rapid development of information technology, the intensity of the flow of capital, goods, services, people, information and other factors has surpassed that of most historical periods. And the “social as society” has been gradually constructed into a “social as mobility” (18). The sociologist Zygmunt Bauman (19) summed up this change in social form with the term “liquid modernity” (19), which suggests that the fluid, indeterminate, and dynamic time-space relations brought about the separation of capital from labor and the shift from a sedentary to a nomadic way of living. Manuel Castells (20), discussing the “rise of the network society”, points out that our society is constructed around mobility, which is not only an element of social organization, but also a manifestation of the processes that govern our economic, political and symbolic life (20). The network has become a fundamental component of the reinterpretation of territorial space. These discourses constitute and drive the notable “mobility turn” in social science research, and “mobility” has become a key term for many scholars to describe and capture the features of contemporary society and the characteristics of the times. How to realize the “re-embedding” of social relations in the modernity of high-speed mobility and how to transcend geographical and social boundaries to achieve relationship expansion, resource acquisition and subject competence enhancement has become the core issue of contemporary social science research.

The reconstruction of social relations, the assimilation of regional culture, and the transformation of power fields closely link “space” and “mobility”. Spatial theory provides an appropriate analytical framework for studying the urban assimilation of migrant elderlies following their children. Taking spatial theory as the research framework, this paper studies the urban assimilation of migrant elderlies following their children from three dimensions: physical, social, and spiritual space, and explores the factors that affect the urban assimilation of migrant elderlies following their children and the necessary measures to make them “feel at home” in in-flow cities.

### Urban assimilation of migrant population

Social assimilation is a classic sociological topic that originates from studies on ethnic immigration in the United States and social integration in Europe, and aims to explore how immigrants from different cultural backgrounds can dissolve cultural differences in order to reach cultural consensus (21, 22). Their research focuses on analyzing the identification mechanisms of immigrant cultures. Western researchers have developed a series of influential social assimilation theories (23), such as the melting-pot theory, segmented assimilation theory and selective assimilation theory, and explored a number of indicators for examining the social assimilation of migrant groups from economic, political, and cultural dimensions. Some representative models are Gordon's (24) two-dimensional model of structure and culture (24), Junger-Tas's (25) three-dimensional model of structural, socio-cultural and political-legal integration (25), and Entzinger's (26) four-dimensional model of socio-economic integration, political integration, cultural integration and attitudes toward migrants in in-flow areas (26). The aforementioned studies have laid the foundation for subsequent explorations on the social assimilation of migrant groups. However, due to the institutional and cultural differences between China and the West, it is difficult for Western societies to find a comparable object that matches the actual situation of China's migrant population. Chinese scholars have combined traditional social assimilation theories with Chinese realities and put forward several influential domestic theoretical hypotheses, such as the “re-socialization” hypothesis which covers economy, society, psychology and culture proposed by Tian Kai (27), the “assimilation progression” theory proposed by Zhang Wenhong and Lei Kaichun (28), and the economic, social, cultural and identity “assimilation interaction” theory proposed by Yang Juhua (29). The subjects of these studies mainly involve migrant workers. Recently, studies on migrant population of ethnic minorities, migrant females and migrant elderlies following their children have gradually increased as other migrant groups become known to wider public.

To be specific, studies on social assimilation of migrant elderlies following their children reveal that, unlike the retirement migration in the west that results from rational-thinking and independent choice (30, 31), the migrant elderlies following their children in China are most passive migrants. In most cases, they migrate for promoting family development and raising their grandchildren. Therefore, family assimilation is the basis of urban assimilation of China's migrant elderlies following their children. Some researchers use the common framework of social assimilation of migrant population to analyze the urban assimilation of migrant elderlies following their children. They suggest that compared to other types of migrant population, it is more difficult for migrant elderlies following their children to realize urban assimilation and the “people-environment fits”, which may exacerbate such health risks as depression and the lack of subjective happiness (32). The factors that affect their assimilation include not only such macroscopic elements as institutional obstructions and social exclusion (33), but also such microscopic elements as language barriers, loss of active interaction and insufficient learning ability (34). However, while applying the analysis framework of social assimilation of migrant population to examine the features of urban assimilation of migrant elderlies following their children, these studies, more often than not, obscure the special needs of urban assimilation of migrant elderlies following their children, which may lead to inaccurate research findings. Meanwhile, these studies tend to emphasize on indicator measurement and phenomenon analysis, thus neglecting the colorful and diverse stories of urban life of migrant elderlies following their children and failing to gain a deeper insight into their urban assimilation. Therefore, this study focuses on the self-narratives of migrant elderlies following their children so as to present the basic patterns of their urban assimilation from their own perspective.

### Spatial transformations and health

The health of migrant populations has been a key issue of social science research in recent years. Related studies show that compared to non-migrants, whereas temporary migrants were at higher risk of mental problems (35). Ren.et al (36) surveyed 915 migrant workers in two Chinese shoe-making factories and found that 31.7% of Assembly-line migrant workers were clinically depressed (36). As the core “mobile” unit in a mobile society, the spatial transformations brought about by the geographical mobility of the population, which have a deeply impact on the health condition of individuals. In physical space, the built environment, land-use patterns, housing conditions can have positive or negative impacts on quality of life and well-being of households and individuals (37). For example, the study by Perdue et al. (38) found that because of lacking adequate safe playgrounds and green spaces built environment of urban space does not promote healthy lifestyles (38), even has a close relationship with the incidence of chronic diseases. Li and Liu's survey of China's mobile population found that poor housing conditions are significantly associated with perceived stress (39). In social space, neighborhoods, social networks, and social support are all closely related to a person's physical and mental health (40). Studies show that social discrimination in the process of social integration of the migrant population not only affects their mental health status and access to health services (41), but has a persistent negative impact on their physiological health indicators such as high blood pressure (42).Social environment formed by neighborhoods, social networks significantly predicts both perceived stress and mental health (39).These studies argue that when promoting the social integration of immigrant groups, the government should not only focus on the geographical compartmentalization of policies, but on developing the social skills of immigrant groups.

For the elderly who move to the city with their children, migration first brings spatial transformations, which in turn brings the need for social integration. Specifically, they are detached from their original living field, dis-embedded from their established social relationships, separated from their acquaintance society, and enter a new space that is physically, socially, and spiritually unfamiliar. So they have to reconstruct their social capital, reshape their value system, and adapt their lifestyles and behavior patterns, re-integrating and integrating themselves into the new territorial space. In the process of social integration, the dilemmas faced by the migrant elderlies following their children and the behaviors of self-motivated adaptation to the new space, which are directly or indirectly related to their physical or mental health. Through a review of previous studies, we can find that previous studies on social integration and health issues of migrant populations, especially migrant elderly, have mostly focused on quantitative correlation analysis, ignoring the rich and diverse life stories of migrant elderly during the migration process, and failing to gain a deeper insight into their urban integration process and its impact on individual health. Therefore, this study takes the self-narratives of migrant elderlies following their children as the main research material under the perspective of spatial theory, presents the basic patterns of their urban integration from the standpoint of migrant elderlies following their children, and explores the impact of urban integration dilemma on the health of migrant elderlies following their children. The research questions are as follows:

1) What kind of urban integration needs do the spatial transformations brought about by mobility create for the migrant elderlies following their children?
2) What kind of integration difficulties do migrant elderlies following their children encounter in the process of urban integration, and how do these difficulties affect their physical and mental health?
3) How to bring into play the subjectivity of the migrant elderlies following their children to solve the integration dilemma, which they face and the derived health problems.

## Research methods and data collection

The authors chose the family residential area of the university (University C) where they are working in as the research site to collect data. The university is located in Wuhan, Hubei Province, China, and its main campus has three residential areas for staff and their families, with which, accordingly, three gathering areas are formed for migrant elderlies following their children. Compared to other communities, the composition of the university's family residential area is relatively simple, consisting mainly of the university's staff and their families. As a “double first-class” university in China, the staffs of University C all over China, and their families are not local residents in Wuhan. So, the selection of research subject can be representative.

After preliminary observation, the researcher finds that 9:30–10:30 a.m. and 7:00–9:00 p.m. are the time when the migrant elderlies following their children in the three residential areas do outdoor activities together. To be specific, 9:30–10:30 a.m. (after breakfast and before lunch preparation) is their free time and many preschool children need to do outdoor activities during this period of time. Therefore, those migrant elderlies following their children will take this opportunity to gather in their community for outdoor activities. And 7:00–9:00 p.m. is the time for children to do outdoor activities after dinner. The migrant elderlies following their children will take their kids to nearby playgrounds. Therefore, they will also get together for recreation. In addition, for some families, this period is also the time the young people take a break from daily work. Many young couples will take care of their children during this time and the migrant elderlies following their children will get the opportunities to enjoy their “vacation” and recreational activities. The most typical recreational activity is “square dancing”. In university C, there are two fixed sites for square dancing around the residential area of migrant elderlies following their children. And they even have WeChat groups to facilitate daily communication.

Given the diversified life of the migrant elderlies following their children, this study adopts the participatory observation and semi-structural interview, so as to gain a better understanding of the life, socialization and local participation of the migrant elderlies following their children in University C. Participatory observation refers to researcher's participation in the gathering activities of the migrant elderlies following their children, including offline participation in the group chatting, square dancing and even taking the kids out for fun together with them. The researcher also joins two square dancing WeChat groups to do close observation and records of the socialization and recreational activities of the migrant elderlies following their children. The researcher has conducted the survey for two months since the study began in May 2022 until July 20, 2022. During this period, the researcher takes field notes to record the patterns and features of behavior of the migrant elderlies following their children to facilitate the subsequent study. In addition to focusing on socialization and recreational activities, the researcher also conducts semi-structured interviews with 15 migrant elderlies following their children (the sociodemographic profile of 15 interviewees are provided in Table 1), which covers multiple indicators such as health care system, urban identify, culture identity, sense of belonging in the city and psychological conditions.

**Table 1:** Sociodemographic profile of 15 interview.

Age categories	55–68 years
Gender distribution	Four men and eleven women
Outflow area	Four from the small city, eleven from the countryside

After the materials were collected, the researcher used NVIVO12 to code the qualitative data. The software has been used by a large number of researchers. They used it to code data for qualitative studies. First, the researcher used open coding, which could initially categorize the interview texts and observation notes in an abstract way, and then used the spindle coding to refine the open coding results again, integrated the social assimilation status and health status of the the migrant elderlies following their children into different spatial categories, it facilitated subsequent analysis.

We maintained standard ethical protocols to conduct this research. The study protocol was approved by the academic committee of the School of Journalism and Cultural Communication of Zhongnan University of Economics and Law. At the beginning of the interviews and observation, informed consent was sought from each participant after a briefing about purpose of the research was done. The identities of the respondents were kept confidential, all the names of the interviewees are replaced by English letters in this paper. We assured that the information provided by the participants would only be used for academic research.

## Research findings

This study explores how the migrant elderlies following their children adapt to the spatial transformations brought by geographical movement, and how such spatial transformations influence the urban assimilation of the migrant elderlies which further affects their sense of belonging in the city and their psychological perceptions of their city life. Under the framework of spatial theory, this section explores the urban assimilation of migrant elderlies following their children in three dimensions: physical space, social space, and spiritual space.

### Adaptation differences in physical space and health risks of migrant elderlies following their children under the urban-rural dual structure

The physical displacement from the outflow area to inflow area is accompanied by a changing of the space in different ways. So, the first change is the physical space, where it is the initial space to which the migrant elderlies following their children are exposed after entering the inflow area, and it is also the type of space that directly affects their life styles and physical health conditions. It mainly involves the area of the city, the climate, and the physical environment of living. This is directly related to the city pattern of the inflow city. Due to the “urban-rural dual structure” in China, the lifestyle and living environment in rural areas are fundamentally different from those in urban areas, and in the process of urbanization, the development between cities are also distinctively different. In terms of population size, economic indicators and modernization level, the indicators of developed cities in China are much higher than those of small and medium-sized cities. As a new first-tier city in China's urban hierarchy, Wuhan has a resident population of 13.649 million, and its economic development and modernization of urban governance are among the leading levels in China. Wuhan is also characterized by cold winters, hot summers and four distinct seasons due to its north subtropical monsoon climate. These external urban conditions affect the integration of the migrant elderlies following their children, in some ways, it even poses a health risk for the migrant elderlies following their children.

Firstly, in terms of natural space, urban-rural and regional differences in climate, which affect the urban integration of the migrant elderlies following their children. Relatively speaking, elderlies who migrated from Hubei Province and neighboring provinces in Hubei have a higher degree of adaptation to Wuhan's climate. Unlike other elderlies who migrated to Wuhan, complaining about the “cold winter and hot summer”, these elderly people can basically accept Wuhan's winter climate. However, with the influence of urban effects and other factors, some of the elderly people who have moved in from other parts of the province also said in interviews, “Wuhan is hot in the morning, midday and evening, in the countryside, it is much cooler at night, and I am particularly unaccustomed to the summer in Wuhan”.

The lack of adaptation to the climate directly adds difficulties to the urban assimilation of the migrant elderlies following their children, even directly affecting their health. As Ms. G, an interviewer from northwest China, says, “*I've been in Wuhan for four years, but I still can't stand the weather there. In my hometown, the maximum temperature in summer does not exceed 33 degrees, and there is a big temperature gap between day and night. It is very comfortable to live in a heated room in winter. But the summer in Wuhan is really uncomfortable, and the winter is the same. I have urticaria because I'm not used to the weather in Wuhan. So, as soon as my grandson goes on vacation, I take him back to hometown*.”

Secondly, in terms of the scope of urban space, most of the migrant elderlies following their children in the family residential area of University C come from small cities, counties, towns and other less densely populated “acquaintance society”. Because the geographical scope is limited and they live in those places all year around, it is unlikely that they will get lost. And the situation when they don't know how to travel will not happen. However, these situations often happen in inflow areas. Especially for the migrant elderlies following their children who migrate from rural areas, it is more difficult for them to travel independently in the bustling city, which limits the possibility of participating in local practices and reinforces their dependence on family members.

“*I have never received education and I cannot read. In my hometown, I know all the roads. However, I dare only to move around the campus now. Wuhan is such a big city. I'm afraid to travel far away by myself because I don't know how to take public transportation and I'm afraid of getting lost. There are times when I get nervous waiting for a traffic light because there are so many cars and I am afraid of getting hurt or violating traffic rules*.”

Interviewee Ms. A spoke frankly about the negative impact of the size of the city on her local participation. She thought that modern urban space has limited her range of activities, making it more difficult for her to get around. Similar to Ms. A's difficulty in traveling around, Mr. W, who moved from a small city, used the phrase “*instantly becoming a fool once arriving in this big city*” to tease his own city life. He says that in the face of Wuhan's complex traffic environment, he cannot drive, and if he wants to travel far away by himself, he has to ask his children to find out the bus route in advance.

Moreover, the development of information technology has promoted the construction of smart cities and has substantially improved working efficiency and speed in various industries. However, its penetration in urban life has greatly affected the urban adaptation of migrant elderlies following their children. For example, the use of electronic smart systems in medical institutions has made it necessary to register, examine, collect reports and take medication through “all-in-one machine”. However, the low level of adaptation to new technologies of the elderly population has hampered their basic life in the city, such as medical care and shopping, which makes them to extremely miss their hometown.

“*In Wuhan, if I go out to buy vegetables or take a bus, I have to pay by cell phone. During the COVID-19 pandemic, I even have to scan the code, which is really troublesome. Sometimes, when I'm in a hurry, I'm not even able to operate my phone. I still think it's good to live in my hometown, there is no such trouble, you can use cash for everything*.” (Interviewee Ms. G)

This discomfort with the scale of modern urban space, as well as the complex transportation environment and technological equipment, directly affects the “sense of efficacy” of urban life for the migrant elderlies following their children, and reinforces their self-perception of being “strangers in urban space”. More importantly, the dilemma of access to health care caused by the electronification of health care, which may affect the timing of medical care for the elderlies and increase their health risks.

Lastly, in terms of living space, most of the respondents in this study are from townships, and their living space before migration is mainly “ quadrangle” type house, with activity areas in the courtyard and intervals between houses, so that they can meet friends when they go out. In the urban space of Wuhan, the living space is dominated by high-rise buildings, and one has to go downstairs for outdoor activities. Therefore, the first thing Ms. A, the interviewee, mentioned when referring to the discomfort of city life was that the “tube-shaped” urban commercial apartment limits the freedom of living.

“*When I live in my rural house, with nature at my doorstep and a yard at home, I can do whatever I want. But in Wuhan, I spend most of my time inside the apartment building. If I want to sunbath I have to go downstairs. There is no way to feel the nature directly, I am bored and lonely. Unlike rural areas, each household is separated by a courtyard, it is easy to disturb each other when there is any noise*.”

It can be seen that the change of living space under the urban-rural dichotomy has changed the lifestyle of the elderly people. They are confined to the living space of commercial apartment and have to be careful not to disturb their children at all times, and their exercise has been greatly reduced as a result, creating a lifestyle that is not conducive to health.

### “Invoking” and “pushing back” of social space transformations for migrant elderlies following their children

The second change is the social space, which is not only products of purposive practice, but also affects space practice of actors. As a type of space that embodies institutional hierarchies and forms of public life, social space can be further divided into institutional space and network space. These two types of space are important elements in classifying and categorizing social individuals, and they profoundly affect the urban assimilation and individual health of the migrant elderlies following their children.

#### “Spatial boundaries” of the migrant elderlies following their children prescribed by the basic medical insurance system

Compared to young people, elderly citizens have poorer health and greater need for medical resources as they age and have more underlying conditions. In the process of urbanization, high-quality medical resources continue to cluster in large cities. Large cities are significantly better than small and medium-sized cities in terms of both the technical level of medical care and hospitalization capacity. “Going to big cities for better medical treatment” once was the preferred choice of the general public in small and medium-sized cities when they were sick. Therefore, it is reasonable to assume that the level and convenience of medical services should be significantly improved for migrant elderlies following their children who move to big cities from small and medium-sized cities or even rural areas. However, in the research process, the interview of the migrant elderlies following their children showed totally different scenario.

“*Last year, I had severe back pain and couldn't straighten up at all, so my son took me to the Union Hospital to have a check. I was shocked to see how much I spent that day just for the checkups and medication. I will definitely never see a doctor in Wuhan again, because I can't get reimbursed at all*.”

Ms. B, who moved to Wuhan from a village in Yichang, Hubei Province, has a new type of rural cooperative medical insurance within China's basic medical security system. The procedures for remote reimbursement of this insurance within Hubei region are complicated, requiring filing at the place of registration and choosing a designated hospital for medical treatment, and direct settlement of outpatient treatment is not available for the time being. The hospitalization procedures in Wuhan's class-A tertiary hospitals are very complicated, and it is difficult to be directly hospitalized for minor illnesses and pains, which directly affects their choice of medical treatment, so “going home for treatment” has become their preferred way of medical treatment.

“*Common diseases such as lumbar spine and rheumatism can be treated in my hometown. It's easy to get hospitalized there, and many physical therapy costs can be reimbursed directly through hospitalization costs. In the future, I will go to the hospital in my hometown to treat these illnesses*.” (Ms. B)

Like Mrs. B, in the investigation, many interviewees pointed out the limitations of remote medical insurance reimbursement in inflow areas. Some migrant elderlies following their children with underlying and chronic illnesses even will purchase a large amount of common medications when they return to the outflow area and take them to the inflow area. However, this type of access to care is likely to lead to delays in treating some of the underlying conditions of the elderly who move with them, thus posing health risks.

Lamont (43) points out that symbolic boundaries exist in various specific social contexts, political forces, institutional power, and cultural resources are constantly generating symbolic boundaries and classifying or categorizing individuals in society accordingly (43). In the same way that the household registration system divides the urban resident population into “local” and “migrant” populations, China's basic medical insurance system is constantly constructing boundaries between “local” and “migrant” populations. The medical insurance system, which is supposed to ease the medical burden of the elderly population, has reduced the compliance of the migrant elderlies following their children to receive medical services due to different settlement ratio of medical services, and also reinforced the perception of the migrant elderlies following their children as “drifter” and limited their urban assimilation process.

However, the supplement of commercial insurance for Chinese basic medical insurance system fills the gap caused by reimbursement for medical treatment, thus promoting urban assimilation of migrant elderlies following their children. For example, the interview of Ms. E shows the impact of “commercial insurance” on her urban life.

“*I bought new rural cooperative medical insurance in my hometown. But since I have hyperlipidemia, hypertension and hyperglycemia, after I came to Wuhan, my daughter-in-law bought me a commercial insurance for my chronic diseases. In this way, even if I don't use the new rural cooperative medical insurance, I can still get 40% reimbursement for medical treatment in Wuhan. The medical conditions in Wuhan are much better than those in my hometown, so I am still willing to seek medical treatment in large hospitals in Wuhan*”.

#### Embedding and pulling back: The dual role of the new social relationship

Cross-regional mobility of population is a process of “deviation of locality” and “dis-embedding”, which implies both the transcending of the original geographical space and the detachment of social relations from the territoriality of mutual interaction (44). Although the migrant elderlies following their children who move from the “acquaintance society” in the countryside and small cities to the family residential areas of Wuhan University C in Wuhan can enjoy reunion with their children in the inflow area, the forced breakage of other social relationships makes them feel lonely when they have just entered the inflow area. The migrant elderlies following their children would use some common phrases to describe their living conditions and psychological states when they have just entered urban space: “*Every day goes by so slowly that I'm almost counting every minute*”; “*I don't know anyone else when I go out, so I have to stay at home all day and I feel that I am in a prison*”; “*It feels like a fishbone getting stuck in my throat and I almost stifled*”. Rebuilding social ties and regaining the “humanity affection” of the traditional acquaintance society in the inflow area has become an important way to relieve loneliness and seek a sense of belonging and identity.

During the research, the author finds that all the rebuilding of social ties of migrant elderlies following their children in the family residential area of University C is almost related to other elder seniors in this community. Almost every day, at a specific time, the migrant elderlies following their children will get together in a “fixed” site of the community. Generally speaking, from 9:30–10:30 a.m., they are surrounded by their grandchildren, while from 7:00–9:00 p.m., some of the migrant elderlies following their children will be freed from the confinement of “raising grandchildren” and form square dancing teams with other seniors to build up their body. During the interviews, when asked about the process of getting acquainted with other migrant elderlies following their children, the interviewees basically said that “it is a natural process”.

“*I came to Wuhan to help my daughter with her kids, and when the kids go out every day to sunbathe and play, I have to go out with them, so I can naturally come in contact with other seniors. Children like to play with children, we old people will also chat. The more times we chat, the more we will naturally get acquainted*”. The interviewee, Mr. I, and his wife have come to Wuhan to help their daughter raise their children for 3 years, and he says that the friends he meets in Wuhan are all migrant elderlies following their children in the community.

Because they share the same life patterns, interactions between the migrant elderlies following their children can be called a “typological” result of “group-individual association”, where they share a common language, face the same social and life problems. It will bring the possibility of intergroup support. The interviewee, Ms. F, has moved to Wuhan for 5 years, and she has known a relatively large group of migrant elderlies following their children, and has become “close friends” with 5 migrant elderly. They often help each other and go on trips to nearby scenic spots together in their free time. “*We often talk about where we can buy cheap vegetables and fruits. Occasionally, when we are too busy, we can leave the children to other elderly people because we all live in the same neighborhood and it is very reassuring to ask them to help*”. Similar interactions and mutual support activities increase their sense of “control” over life,reducing the feeling of loneliness in unfamiliar spaces, and the frequency of participation in urban life. When interviewed, Ms. F says, “*Knowing other migrant elderlies following their children has given me a sense of happiness, and they are an important reason why I can stay in Wuhan for a long time*”.

In the daily interactions among migrant elderlies following their children, there is a special way of interaction, namely “square dancing socialization”. Studies in the field of journalism and communication in China have pointed out that square dancing is an important way to rebuild interactions and reconstruct identities among the elderly in today's China. This is also true for the migrant elderlies following their children. There are two specific square dancing sites for the migrant elderlies following their children of three family residential areas in University C. When the task of taking care of grandchildren is undertaken by their adult children, the migrant elderlies following their children will get together in the two sites from 7:00–9:00 p.m. everyday to exercise by square dancing. During breaks, they will get together for a casual chatting. In recent years, with the penetration of smart phones and social media in the lives of the elderly, WeChat groups have also become popular among the migrant elderlies following their children. In order to enhance contact, spread messages of square dancing and stimulate each other to keep exercising, the two square dancing groups in the family residential area of University C have also created their WeChat groups. The existing square-dancing communities in the real space are transformed into a network, which is transplanted, maintained and extended in the cyberspace, thus forming an important space for them to interact and communicate with others in the inflow areas. More importantly, this interaction space enhances the psychological comfort of the migrant elderlies following their children at the same time, becomes an important tool to monitor their exercise, which is beneficial to their healthy life.

During the two months when the author has been observing the two square dancing groups, the two groups have new messages every day. The information content involves ritualized greeting messages, the release of square-dancing instruction videos, advice on household and the sharing of daily life. Occasionally, there are even topics that are difficult to discuss with children in the family space, such as ‘how to spend our old age, whether we should return to our hometown in the future” and “the restrictions imposed by children on our lives”. For the migrant elderlies following their children, interacting with others in WeChat groups has become an important “pastime” and “comfort” for them to spend the repetitive life after finishing household chores. As Ms. F says in the interview, “*Other migrant elderlies following their children will post videos of cooking and children playing in the WeChat groups. If I chat with them when I am free or bored, I will feel better and time would go by faster*”.

It can be said that social media, represented by WeChat, has extended the social relationships of the migrant elderlies following their children in the real space through instant interaction, and made them more stable and closer. The “emotional comfort” function of interpersonal communication among the migrant elderlies following their children is also enhanced because of the information and communication technology.

Unlike the intergroup relationships in the inflow areas, which can promote the embedding of the migrant elderlies following their children into urban life, social media's “reconnection” to social relationships in the “outflow areas” of the migrant elderlies following their children causes a “pulling-back effect” on urban integration, limiting the migrant elderly's sense of belonging to the city. Before migration, the lives and social relations of migrant elderlies following their children are constructed over a long period of time and with a high level of territoriality. It is rooted in the network of human relationships and acquired normative knowledge in their hometowns, and is an important source of their sense of practice, belonging, and individual security. However, the mobile life has broken the entire set of social ties they used to have. The original social relations based on hometown and occupation have become difficult to maintain, and the homeland has become a “faraway home” to a certain extent. However, the intermediation of the Internet and mobile technologies has made “portable communities” possible (45). Therefore, mobile people can maintain social interactions and realize emotional connectedness in their original physical space through the Internet, whether they meet or not. This makes the community free from the physical space and become “portable”. In order to keep in touch with their friends and relatives in the “outflow” area, most of the elderly who migrate to the family residential area of University C have the WeChat of their friends and relatives in the outflow area, and a small number of the elderly who do not use social media also maintain their past social relationships through phone calls. The life style of their hometown is clearly presented in front of them through social media, invoking and deepening their memory and emotion of the “outflow place”.

Mr. D, who moved to Wuhan from Dazhou, Sichuan Province, was a village cadre in his village before moving to Wuhan, and after coming to Wuhan, he still follows various issues in his village through social media, and even acts as a “mediator” through social media when he hears about the conflicts in his village. “*I am now also very close to the villagers. A neighbor once took a picture of my old house and sent it to me, and I was extremely eager to go back*”.

Homesickness is very common among migrant elderlies following their children, especially for those who move to Wuhan alone. Their spouses still live in their hometowns. When watching these videos, their “desire” for “returning hometown” will grow even stronger. Mr. D even said bluntly in the interview, “*We will definitely return home to spend our old days. We need to contact our friends and relatives in the hometown more frequently, so that it is convenient to go back. Friendships in Wuhan will not be as important as those in the hometown in the future*”.

It can be concluded that although social media, represented by WeChat, have made social networks and physical patterns of “outflow area” “portable” and “viewable”, and they have also, to a certain extent, limited the urgency and enthusiasm of social relationship construction in the inflow area, thus slowing down the generation of sense of belonging in the city.

### The pull-back effect of “family view” and the mental health of migrant elderly

In addition to physical presence, space is a social and conceptual construct^6^. Geographical mobility is not merely “relocation”, but is accompanied by changes in spiritual or cultural space. So, the third space is spiritual space, which is the decoding of cultural symbols, the identification of values, and the sense of belonging are all indicators of the reference to. When migrant elderlies following their children move from their familiar living space to the city where their children live, they are first exposed to and involved with the families their children have established in the city. The perception of their children's families directly affects their self-identity orientation and their sense of belonging to the city, as well as their behavioral norms and acquisition of urban culture.

Chinese traditional concept of clan and child-bearing have always emphasized the importance of “extended family”. The most promising vision of family life used to be “children and grandchildren pervading the hall” because Chinese people used to think it's blessed to have a lot of offspring. However, with the development of urbanization and changes in social life, the family, which is the “origin” of individual existence, is also changing, and the family form is shrinking. The widely spread “nuclear family” among young couples excludes the presence of grandparents in the construction of the meaning of “family”. However, in contradiction to the construction of the meaning of small families, the widespread phenomenon of “grandparents raising their grandchildren” in China shows the diversity and complexity of Chinese families in a substantive sense. Some studies use “Neo-familism” (46) to explain the new multigenerational family model that has emerged in China after 2000. They think such familism results from the pursuit of individual happiness and family wealth through intergenerational synergy, and it places family interests above individual interests. In this family model, the elderly constantly weakens individual rights and interests to ensure that the interests of the young couples are maximized. From the perspective of behavioral and emotional orientation, the migrant elderlies following their children consider themselves to be part of the “family” with their children and grandchildren. However, the researcher also finds that most of the migrant elderlies following their children do not consider themselves as part of their children's families in the inflow areas because of their homeland affection and because they consider the new living space established by their children in the city as an important sign of their children's individual accomplishment. For example, Mr. W, an interviewee, said bluntly during the interview, “*This is the home of my son and daughter-in-law, not my own, and I am here only to help them. This isn't my home, and I will definitely go back in the future*.”

There are many migrant elderlies following their children who share Mr. W's view of family. Under the influence of this family view, they position themselves as “temporary residents” in the urban space and insist that “the outflow area” is their ultimate home, so they are not interested in learning Wuhan dialect and cultural customs, which limits their spiritual assimilation in the inflow area, it also reinforces the “loneliness” of their life in Wuhan. Ms. H, who moved to Wuhan from Henan for 3 years, said that she still can't understand Wuhan dialect. “*I rarely talk with Wuhan locals, and I don't want to learn Wuhan dialect because it's not necessary. In a few years, when my grandchildren grow up and go to school, I'm going back to Henan. Although it is true that I will feel lonely, but it will be better when I go back to Henan.”*

In addition, under the influence of the concept of “small family”, it is difficult for migrant elderlies following their children live with their sons and daughters-in-law to truly integrate into the family space in the place they move to. On the other hand, they feel that their sons and daughters-in-law are family members and that they can only play the role of “helper” and cannot make any important decisions in their small families. Therefore, “listening to my son and daughter-in-law and obeying their arrangements” is the code of conduct for family life in their self-reporting. In this context, some of the migrant elderlies following their children seldom make voice about their demands in the family space, and their subjective recreational and leisure needs are hardly satisfied, which to a certain extent reduces the sense of comfort in the life of the elderly. At the same time, due to the differences in living habits and child-rearing concepts, the elderly living in the same physical space will always have conflict points with the younger generation, while the conflicts between mothers-in-law and daughters-in-law, which are not mediated by blood relations and emotional bases, can easily be intensified, thus negatively affecting the psychological health of the elderly migrants. As Ms. K said in the interview.

“*My daughter-in-law and I both have strong personalities, and we both often have conflicts because of bringing up children, although we don't really hold grudges, but frequent quarrels do affect the mood, and when the children are a little older, I still have to go back and can't live together all year round.”*

As seen above, the “mother-in-law-daughter-in-law conflict” in Chinese family relationships also plays an important role in the urban integration of migrant elderlies following their children, which affects the length of urban life of migrant elderlies following their children, and may also bring the risk of depression and reduce their sense of wellbeing.

## Discussion

Rapid population aging and unprecedented population mobility have been the main demographic features of Chinese society in the last 40 years. In the new historical context, migrant populations of different generations have also changed. However, unlike the predictions of classical migration and mobility theories, the size of China's migrant elderly is becoming increasingly large, forming a unique normative phenomenon. As an important part of family migration in China, the geographical migration of the migrant elderlies following their children changes the physical, social and even spiritual and cultural spaces they are accustomed to, leading to adjustment and assimilation needs, and to some extent, increasing health risks. In this context, exploring the urban assimilation of China's migrant elderlies following their children is in line with the national reality and can be responsive to the UN Sustainable Development Agenda's call to promote the wellbeing of people of all ages. Unlike the previous studies on social assimilation, this study introduces the spatial theory of sociology and uses it as a framework to analyze the factors affecting the urban assimilation of the migrant elderlies following their children from three aspects: physical, social, and mental space. Theoretically, it is a beneficial supplement and expansion of social assimilation theory, and a practical test of the spatial theory.

This study finds that for the migrant elderlies following their children who are passively involved in the process of family mobility, family assimilation is the foundation of their urban assimilation and the beginning of a stable life in the city. Therefore, harmonious intergenerational relationships, a sound family atmosphere, and family-based urban practices are the basic strategies to promote urban assimilation of the migrant elderlies following their children. Secondly, the urban life of the migrant elderlies following their children is mostly community-based, and their activities are basically confined to the community. It is especially important for them to gradually adapt and assimilate into the new living space and develop a sense of belonging by reconstructing close neighborhood relations and regaining the humanity affection of the traditional acquaintance society. Currently, the horizontal organization of Chinese grass-root communities is poorly developed, and the reconstruction of social relationships of the migrant elderlies following their children basically relies on their own practices. The community does not provide any specific support. In the future, the functions of the community should be proactively explored in the process of promoting the social assimilation of the migrant elderlies following their children. Lastly, the fundamental institutional barrier is an significant factor that influences the ability of migrant elderlies following their children to live a stable urban life. The Chinese government needs to promote a nationwide unified pension and health insurance system, so that the migrant elderlies following their children can enjoy the same benefits as the local elderly residents in the “inflow” area, such as transportation, public facilities, and preferential pension costs. At the same time, the Chinese government needs to strengthen the “accessibility” of urban spaces for the elderly. In addition to infrastructure such as barrier-free facilities, the “accessibility” of modern technology to the elderly in urban spaces should be taken into consideration.

It is worth noting that this study also finds that the penetration of communication technologies into the elderly, represented by the social media, has improved ability of the migrant elderlies following their children to deal with their “drifting” life to a certain extent, and has expanded their social networks. However, its dual spatial connection between the outflow and inflow areas also creates a “two-way pulling” on the urban assimilation of the migrant elderlies following their children.

## Conclusion

China entering a new form of aging society, face urban assimilation difficulties especially for migrant elderlies following their children. With discussion on the spatial framework, this paper explores the factors affecting urban assimilation of these migrant elderlies in a University Community in Wuhan city, Hubei Province from May 2022 to July 2022, researchers participated in their daily life for two months, and conducted 15 semi-structured interviews. The study reveals the following findings: first, family assimilation is the foundation of urban assimilation for these migrant elderlies; Second, urban life of these migrant elderlies is mostly community-based. Third, the fundamental institutional barrier is a significant factor that influences the ability of these migrant elderlies to live a stable urban life. Suggestions for governments to improve the urban assimilation are proposed including promoting a nationwide unified pension and health insurance system.

Due to the limitations of the study field and duration, this study only explores the urban assimilation of the elderly who move to the family residential area of university C. The composition and life patterns of the family residential area of university C are simple, and it cannot fully reflect all the situations of China's urban communities. Future studies could focus on the life patterns of migrant elderlies following their children in diversified communities to explore different aspects of their urban assimilation.

## Data availability statement

The original contributions presented in the study are included in the article/supplementary material, further inquiries can be directed to the corresponding author.

## Ethics statement

The studies involving human participants were reviewed and approved by the academic committee of the School of Journalism and Cultural Communication of Zhongnan University of Economics and Law. The patients/participants provided their written informed consent to participate in this study.

## Author contributions

YB and QL designed the study. YB and JT collected the data. All authors contributed to the article and approved the submitted version.

## Funding

Supported by the basic scientific research project of Zhongnan University of Economics and Law (No. 31512210305).

## Conflict of interest

The authors declare that the research was conducted in the absence of any commercial or financial relationships that could be construed as a potential conflict of interest.

## Publisher's note

All claims expressed in this article are solely those of the authors and do not necessarily represent those of their affiliated organizations, or those of the publisher, the editors and the reviewers. Any product that may be evaluated in this article, or claim that may be made by its manufacturer, is not guaranteed or endorsed by the publisher.
